# Turkish Older Workers in an Emerging Pensions and Retirement Landscape

**DOI:** 10.1093/geroni/igaf122.1437

**Published:** 2025-12-31

**Authors:** Loretta Platts, Sima Karakullukçu Erdem

**Affiliations:** Stockholm University, Stockholm, Stockholms Lan, Sweden; Stockholm University, Stockholm, Stockholms Lan, Sweden

## Abstract

Pension claiming and retirement behaviors in middle-income countries offer rich insights for studies on the economics of ageing. In recent decades, Türkiye has experienced rapid urbanization, industrialization, and formalization of its economy. Its contributory pension funds have developed within living memory. Pension eligibility is based on days of registered employment, gender and cohort; chronological age is largely irrelevant. Interviews with 23 working older adults living in north-west and north-east Türkiye explored pension saving, economic strategies in later life and the meaning of retirement. Translated transcripts were analyzed using constant comparative analysis, in which categories were described and refined by writing analytical memos. Pensions generally played a minor role in safeguarding economic security in later life; lifestyle changes and rental and labor income were more central. Generally, pensions were low and viewed as providing extra income from mid-life, rather than heralding a retirement phase. An image of a leisure-filled retirement was generally absent, instead participants expressed a strong work ethic, in which working is what humans do and how one lives in a society with others. Retirement involved risks of idleness and unproductivity. Pensions are gradually becoming part of the taken-for-granted institutional set-up in Türkiye: in discussing their children’s pensions, participants expressed a growing acceptance of paying premiums and trusting pensions as a reliable income source in a capricious economy. Ceasing paid work—with no link to chronological age and little link to pension claiming—was highly individualized, a direction in which high-income countries may also be headed.

